# Biologic activity and treatment resistance to gastrointestinal cancer: the role of circular RNA in autophagy regulation

**DOI:** 10.3389/fonc.2024.1393670

**Published:** 2024-08-30

**Authors:** Bo Zhang, Zhe Li, Guoliang Ye, Kefeng Hu

**Affiliations:** ^1^ Health Science Center, Ningbo University, Ningbo, China; ^2^ Department of Gastroenterology, The First Affiliated Hospital of Ningbo University, Ningbo, China

**Keywords:** autophagy, biological activity, circular RNA, drug resistance, gastrointestinal cancer

## Abstract

Circular RNAs (circRNAs) lack the 5’-end methylated guanine cap structure and 3’ polyadenylate tail structure, classifying it as a non-coding RNA. With the extensive investigation of circRNA, its role in regulating cell death has garnered significant attention in recent years, establishing it as a recognized participant in cancer’s biological processes. Autophagy, an essential pathway in programmed cell death (PCD), involves the formation of autophagosomes using lysosomes to degrade cellular contents under the regulation of various autophagy-related (ATG) genes. Numerous studies have demonstrated that circRNA can modulate the biological activity of cancer cells by influencing the autophagy pathway, exhibiting a dualistic role in suppressing or promoting carcinogenesis. In this review, we comprehensively analyze how autophagy-related circRNA impacts the progression of gastrointestinal cancer (GIC). Additionally, we discuss drug resistance phenomena associated with autophagy regulation in GIC. This review offers valuable insights into exploring potential biological targets for prognosis and treatment strategies related to GIC.

## 1 Introduction

### 1.1 CircRNA

**Figure 1:**
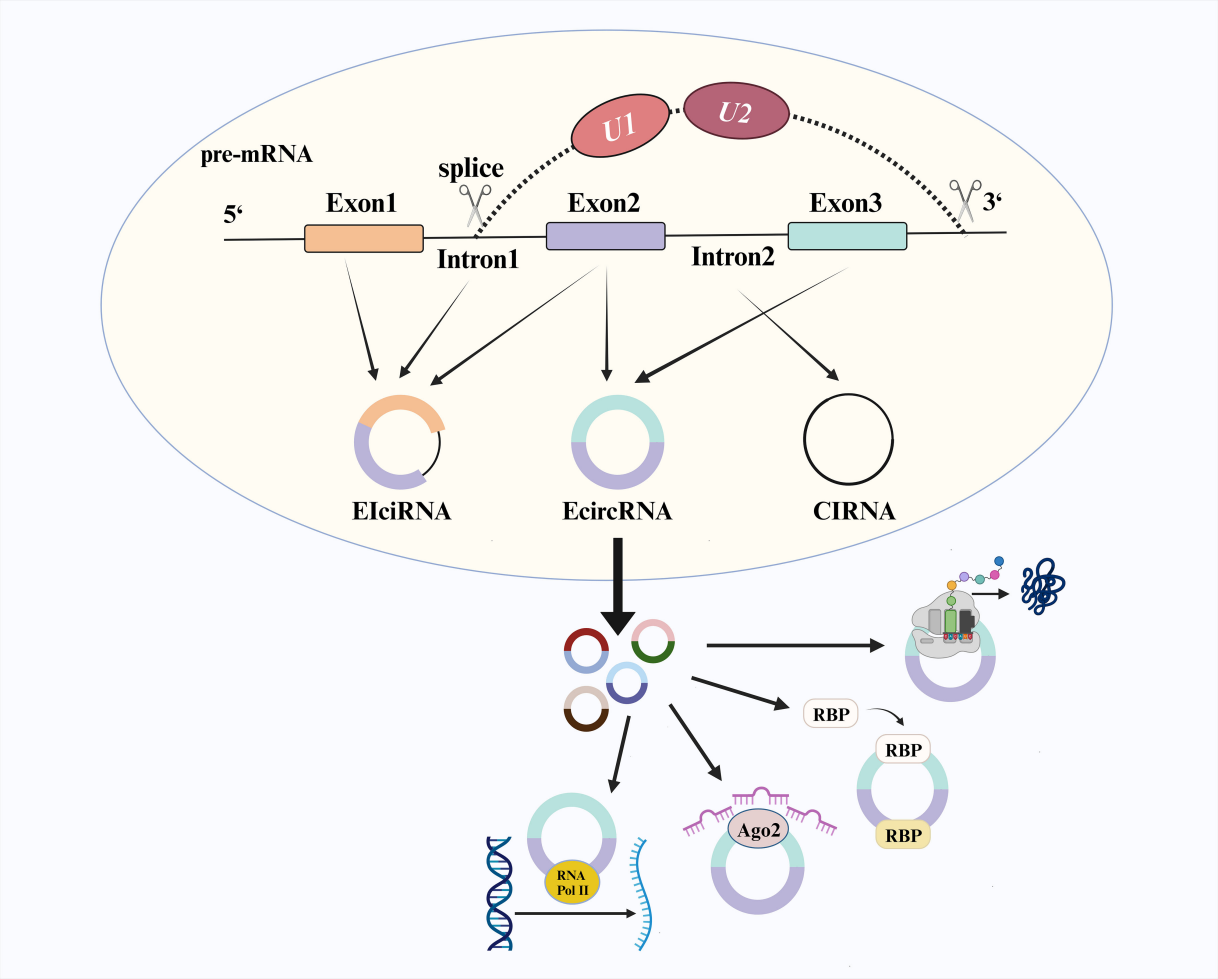
The biogenesis and function of circRNA.

CircRNA is a covalently connected closed-loop structure formed by the reverse splicing protein-coding genes in eukaryotic cells (1). Most circRNA consists only of exons, known as exonic circRNA (EcircRNA). At the same time, some retain intron composition and are referred to as circular intronic RNA (CiRNA) and exon-intron circRNA (EIciRNA) (2) (**Figure 1**). The splicing process begins with the selection of splice sites on pre-mRNA under the influence of the canonical splicing mechanism, where U1snRNP (U1) and U2snRNP (U2) play crucial roles in mutual regulation during site selection (3, 4). Previous studies have demonstrated that changes in U2/U1 levels generate different splicing products. Depletion of U2 can inhibit the splicing factor and increase the production level of circRNA (5, 6). Initially considered a non-functional product of pre-mRNA (7), further research has revealed various functional mechanisms associated with circRNA, including transcriptional regulation, formation of circRNA-RNA binding protein (RBP) complex, microRNA (miRNA) sponge activity and translation function. CircRNA with an open reading frame was initially overlooked due to the absence of a canonical start codon and the challenges in detecting encoded peptides. However, recent research has revealed that circFNDC3B exists in colon cancer and encodes circFNDC3B-218aa, which induces FBP1 expression and inhibits the growth and metastasis of colon cancer (8). Simultaneously, global social and economic development has shifted the disease epidemiology model from acute infectious diseases to chronic non-communicable diseases, particularly with an increasing burden of cancer worldwide (9, 10). CircRNA has been found to exhibit differential expression levels in various types of cancers, such as gastric cancer (GC), colorectal cancer (CRC), lung cancer, and leukemia (2). Combining different circRNA may enhance early detection stability for cancers. Compared to linear RNA molecules, circRNA possesses a more stable covalent ring structure that avoids exonuclease degradation and can be widely detected in blood samples, tissues, and saliva samples, among others. Henceforth, circRNA holds significant potential as a novel biomarker for cancer (7, 11).

### 1.2 Autophagy

Autophagy is a crucial form of PCD that maintains cellular homeostasis by degrading or utilizing intracellular harmful or accumulated substances (12). Researchers have gained a more comprehensive understanding of how autophagy impacts human disease progression in recent years through molecular mechanism studies involving knockdowns of ATG genes and analysis of ATG protein products (13).

#### 1.2.1 The biological process of autophagy

**Figure 2:**
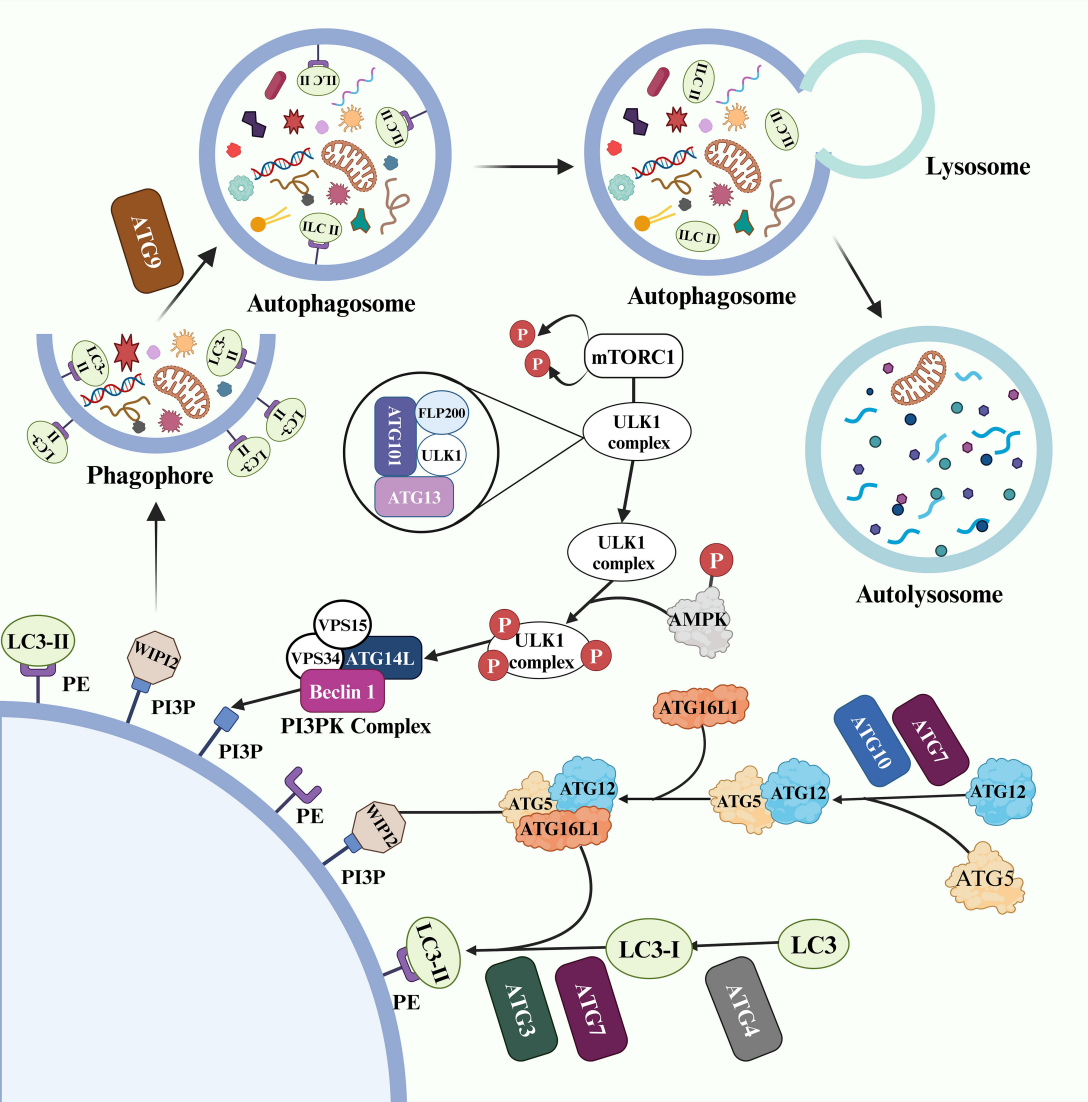
The physiological process of macroautophagy is regulated by a series of ATG.

Three main types of autophagy have been identified: macroautophagy, microautophagy, and chaperone-mediated autophagy. Among these, macroautophagy is a highly regulated multi-stage process (14) (**Figure 2**). ATGs coordinate with each other and selectively recruit core autophagy components to establish double-membrane vesicle structures called autophagosomes. Subsequently, under the influence of effector factors such as soluble N-ethylmaleimide-sensitive factor attachment protein receptor (SNARE) proteins, GTPase, and motor proteins, the outer membrane of the vesicles fuses with lysosome to form autolysosome (15).

In this process, the physiological process of macroautophagy is regulated by a series of ATG (16). The UNC-52-like kinase 1 (ULK1) complex, consisting of ATG101, ATG13, FLP200 (focal adhesion kinase family interacting protein of 200kD) and ULK1, serves as a critical initiator of autophagy and its activity is controlled through mechanistic target of rapamycin (mTOR) complex 1 (mTORC1) phosphorylation and dephosphorylation (17). During starvation conditions, the ULK1 complex dissociates from dephosphorylated mTORC1, increasing activity in the free ULK1 complex (18, 19). Additionally, glucose deficiency activates AMP-activated protein kinase (AMPK), which provides phosphate groups to the ULK1 complex for initiating autophagy (19). The activated ULK1 complex then regulates the phosphoinositide 3-kinase (PI3K) complex composed of ATG14L, Beclin-1 (BECN1), VPS34 and VPS15 that participates in phosphatidylinositol 3-phosphate (PI3P) production from PI on the phagophore membrane to promote autophagy (20, 21). WD repeat domain, phosphoinositide interacting 2 (WIPI2) is recruited by PI3P (22).

Two ubiquitin-like cascade modules modify the early stages of autophagy (23). Firstly, ATG5 and ATG12 bind to ATG16L1 with assistance from two ubiquitinating enzymes (ATG7 and ATG10), forming an ATG5-ATG12-ATG16L1 complex. This complex subsequently binds to WIPI2 and localizes on the surface of the phagosome membrane (24). Moreover, ATG4 cleaves the microtubule associated protein 1 light chain 3 (LC3) termini to generate LC3-I, which participates in the production of LC3-II by co-coupling with ubiquitinating enzymes ATG3, ATG7, and the ATG5-ATG12-ATG16L1 complex on the phagophore membrane (25). Under regulation by ATG9, both the inner and outer membranes of the phagophore expand simultaneously to form a fully closed autophagosome. The cargo to be processed enters the autophagosome through selective or non-selective recognition pathways (26).

BECN1 and LC3-II play critical roles in the process of autophagy and serve as important markers. BECN1 promotes PI3P activation, which leads to the expansion of phagosome membranes and the formation of double membrane vesicles (27). LC3-II recruits ATG2 to interact with WIPI to transport essential lipids for autophagosome formation (28).

#### 1.2.2 Autophagy and cell death

The leading roles of autophagy in dying cells can be categorized into two types: autophagy-dependent cell death (ADCD) and autophagy-mediated cell death (AMCD). ADCD relies solely on components related to autophagy to induce cell death. On the other hand, AMCD triggers various modes of cell death through autophagy, and these two processes can occur independently or simultaneously (29).

Based on specificity, ADCD can be classified into non-selective and selective autophagy (30). When the internal environment detects a lack of nutrients, it initiates the formation of autophagosomes that indiscriminately engulf organelles to ensure sufficient cellular functionality under nutrient-deprived conditions (30). However, current research primarily focuses on selective forms of autophagy, such as mitophagy, ER-phagy, and xenophagy (31). Selective autophagic processes involve receptor proteins with distinct features like LC3-interacting region (LIR) domains and ubiquitin-binding sites that differentiate them from typical selective-autophagy-related proteins (32). For instance, PTEN-induced putative kinase 1 (PINK1) restricts mitochondrial damage by accumulating at damaged mitochondria’s outer membrane through E3 ubiquitin ligase Parkin-mediated ubiquitination; subsequently identified by ubiquitin-proteasome system for degradation leading to PINK1/Parkin pathway-induced mitochondrial-autophagic response (33). Additionally, targeting LIR regions instead of relying on the ubiquitin-proteasome pathway also initiates selective forms of autophagy, an example being TEX264 (an ER-phagic receptor), where its LIR domain interacts with the phagosome, resulting in endoplasmic reticulum clearance under damaged conditions (34).

The emerging evidence substantiates the intricate interplay between autophagy and apoptosis pathways (35); for instance, the crosstalk between PINK1-BECN1 regulates the interplay of autophagy and apoptosis pathways, thereby influencing cancer cell proliferation (36). The convergence of these two signaling cascades is contingent upon the involvement of the second messenger Ca^2+^. Anthraquinone derivatives have been discovered to concurrently induce apoptosis and autophagy in reproductive system tumors as well as digestive tract tumor cell lines (37). Autophagy can also expedite ferroptosis initiation for anti-tumor programming. Erianin, a natural compound derived from Dendrobium officinale, interacts with KRAS^G13D^ mutation to trigger autophagy-dependent ferroptosis while inhibiting the growth and migration of CRC (38). The process of anoikis represents a specific form of PCD that actively contributes to maintaining homeostasis by eliminating cancer cells exhibiting metastatic behavior in the absence of cellular adhesion and loss of extracellular matrix (39). Wu et al. demonstrated that ELAVL1 exerts inhibitory effects on circspecc1 in GC, suppressing of autophagy through ubiquitination and subsequent degradation of ATG4B. Moreover, reduced resistance to anoikis was observed, thereby promoting increased metastasis in GC (40). The mechanism of AMCD, however, remains elusive, and further investigation is warranted for elucidation.

#### 1.2.3 The regulatory factors of autophagy

The increasing body of research has unveiled that autophagy is subject to the influence of diverse regulatory factors (**Table 1**); however, a comprehensive understanding of the precise molecular mechanisms governing autophagy remains elusive (54). Currently, extensive research is being conducted on circRNA’s interference with target gene expression through competitive binding with mRNA via Ago2 sponge miRNA, and its direct or indirect modulation of autophagy efficiency (55). Liang et al. demonstrated that circCDYL acts as a molecular sponge for miR-1275-ATG7, thereby enhancing autophagy in breast cancer (BC) cells by regulating ULK1 expression and ultimately exacerbating the poor prognosis of BC (49). Furthermore, the circRNA-autophagy axis can also regulate chemoresistance to various drugs, thus determining the efficacy of cancer treatment (56). In oral squamous cell carcinoma (OSCC), it was discovered that the circ-PKD2/miR-646/ATG13 axis promotes autophagy and enhances therapeutic sensitivity to cisplatin (DPP) in OSCC (57). Recent studies have also revealed that exosome-loaded circRNA can be transferred between cancer cells, activating WNT-CTNNB1/β-catenin signaling while augmenting autophagy and reducing chemosensitivity to accelerate tumor metastasis (58).

**Table 1:** The role of molecular regulators in the autophagy.

The regulation of autophagy	Regulatory factors	Interaction with the autophagy	Ref
Members of the autophagy signaling pathway	ULK1/2 complex	The ULK1 and ULK2 bind to the FIP200 chaperone proteins, facilitating their interaction with ATG13 and ATG101, respectively. This interaction exposes the LIR motif and is crucial in promoting autophagy during complex assembly.	(41)
	mTOR	The mTORC1 complex is formed, which directly phosphorylates ULK1 at the serine757 site and disrupts the interaction between AMPK and ULK1 in the presence of abundant nutrients, thereby inhibiting autophagy. Additionally, the phosphorylation of mTORC1 negatively regulates autophagy nucleation and expansion.	(42)
	AMPK	In the absence of nutrients, the activity of the mTORC1 complex is diminished, leading to a weakened binding between mTORC1 and ULK1, and subsequent phosphorylation of multiple serine sites on free ULK1 by AMPK, thereby triggering autophagy.	(42)
	PI3PK complex	The complex of ATG14L, BECN1, VPS34, and VPS15 compounds primarily regulates ULK1 phosphorylation at the serine loci of ATG14L and BECN1, respectively. This regulation aims to enhance the activity of ATG14L and BECN1, thereby promoting the formation of the PI3P complex and inducing autophagy nucleation followed by subsequent expansion.	(41)
Post-translational modifications	acetylation	The acetyltransferase EP300/p300 acetylates the K29, K771, and K781 sites of the PI3PK complex, reducing PIK3C3 complex activity. Consequently, it exerts inhibitory effects on autophagy initiation.	(43)
	phosphorylation	The recruitment of p38 kinases to autophagosomes occurs via the Gadd45β–MEKK4-p38 pathway, subsequently leading to the phosphorylation of ATG5 at T75. This phosphorylation event hinders lysosomal fusion and autophagy.	(12)
	ubiquitination/ ubiquitin-proteasome system (UPS)	UPS pathway represents an alternative mechanism for cellular component degradation that operates independently of autophagy. It has been demonstrated that UPS and autophagy are intricately interconnected. The ubiquitin ligase TRAF6 facilitates the ubiquitination of ULK1, thereby enhancing the initiation of ULK1-mediated autophagy by stabilizing its structure and function. Similarly, under the influence of TRAF6 ubiquitination, BECN1 can also promote increased autophagic activity.	(44)
Epigenetic modification	DNA methylation	DNA methylation, catalyzed by DNA methyltransferases (DNMTs), is the most prevalent form of epigenetic abnormality. Inhibition of DNMT3B methylation by lncRNA H19 reduces BECN1 promoter methylation, promoting cell autophagy and attenuating tamoxifen sensitivity in BC.	(12)
Transcription factor	transcription factor EB (TFEB)	Under conditions of sufficient nutrition, mTORC1 phosphorylates TFEB to predominantly localize it in the cytoplasm, thereby suppressing autophagy-related transcription. Conversely, under nutrient-deprived conditions, TFEB is dephosphorylated and translocates into the nucleus, where it binds to the GTCACGTGAC motif on target gene promoters, sequentially activating autophagy-related genes.	(45)
	kruppel-like factors (KLFs)	The expression of KLF10 is significantly higher in human osteoarthritis cartilage, indicating the specific involvement of silent KLF10 in regulating Bcl2-interacting protein 3-mediated mitochondrial autophagy and reducing cytokine secretion. Moreover, it also promotes chondrocyte proliferation and inhibits chondrocyte senescence.	(46)
	transcription factor Nuclear factor-erythroid derived-2-related factor-1 (Nrf1)	The activity of Nrf1 is modulated in response to changes in proteasome number and function. In the event of proteasome damage within the UPS pathway, NrF1-dependent transcription up-regulates relevant autophagy pathway components to enhance intracellular clearance through autophagy compensation.	(47)
Non-coding RNA	long non-coding RNA (lncRNA)	LncRNA FIRRE facilitates autophagy and attenuates the radiosensitivity of endometrial cancer by orchestrating the miR-199b-5p/SIRT1/BECN1 axis.	(48)
	circRNA	The promotion of autophagy, BC cells proliferation, and tumor growth is facilitated by the interaction between circCDYL and miR-1275 targeting the ATG7/ULK1 axis.	(49)
	miRNA	Promoting thyroid cancer cell proliferation and metastasis by miRNA-363-3p is facilitated through the mediation of SYT1 to suppress autophagy.	(50)
	tRNA-derived small RNA (tsRNA)	tRNA-derived RNA fragment (tRF) is classified as a subclass of tsRNA. The inhibition of autophagy by tRF-3001b leads to an increase in lipid synthesis, exacerbating nonalcoholic fatty liver disease pathology.	(51)
Trace element necessary	Copper (Cu)	Cu promotes autophagy in mouse spermatogonia by mediating the AMPK-mTOR axis and increasing the expression of ATGs.	(52)
	Zinc (Zn)	The inhibition of the autophagy process due to Zn deficiency under low Zn diet conditions adversely affects the testis structure and semen quality in mice.	(53)

#### 1.2.4 Autophagy is a double-edged sword

Autophagy is a crucial biological process that profoundly impacts cancer. An increasing body of research has established a strong association between autophagy and the initiation, progression, and drug resistance to cancer. The dual effects of autophagy activation on cancer are captivating (59). On the one hand, within the tumor microenvironment, malignant cells experience rapid proliferation and often encounter hypoxia and malnutrition conditions. Autophagy eliminates intolerable cells while promoting the survival of cancer cells under stressful circumstances, including enhancing their tolerance to drug therapy environments (56). Emerging evidence suggests that ISG15, a protein involved in post-translational modifications, stabilizes ATG7 structure and function while augmenting autophagic flux to counteract gemcitabine (GEM) sensitivity in pancreatic cancer (PC) therapy—a factor associated with poor prognosis in PC (60).

On the other hand, when toxic or malignant substances invade normal cells, autophagy inhibits inflammation, oxidation, and necrosis at the primary site while releasing immune regulatory factors to reduce infiltration by foreign harmful cancer cells (61). Lodder et al. simulated autophagy inhibition by knocking out ATG5 and found that mouse liver cells recruit more monocytes to aggravate liver fibrosis (62). Additionally, autophagy can enhance chemotherapy-induced cytotoxicity, improving drug sensitivity in cancer cells (63). Up-regulation of miR-519a expression was found to increase glioblastoma cell sensitivity towards temozolomide—an effect achieved through promoting autophagy via dissociation of Bcl-2/BECN1 complex as well as inhibiting STAT3/Bcl-2 axis activity (64). The dichotomous nature of autophagy reminds for clinicians to carefully consider whether to inhibit or activate this process during targeted therapies since each scenario yields distinct outcomes.

#### 1.2.5 Therapies targeting autophagy

As the understanding of autophagy in cancer progression mechanisms grows, more autophagy compounds are being developed for future clinical trials and drug screening. WJ460, C150, Oroxylin A, and GNS561 have shown potential in selectively targeting autophagy and in anti-tumor effects in PC and hepatocellular carcinoma (HCC) (31). Tigecycline, commonly used for multidrug-resistant bacterial infections, has also been found to induce selective autophagy and inhibit GC cells activity (31).

The only clinically approved autophagy inhibitor is chloroquine (CQ) derivatives. Clinical trials have reported that CQ and hydroxychloroquine (HCQ) act as autophagy inhibitors by disrupting lysosomes and impairing Golgi apparatus function, particularly interfering with autophagosome-lysosome fusion, thus enhancing the anti-tumor efficacy of chemotherapy drugs (65). However, clinical trials using CQ derivatives for GIC, such as PC and CRC, have yielded mixed results (66, 67).

In recent years, a new class of protein-protein interaction (PPI) autophagy inhibitors based on ATG4 and ATG7 proteins has been developed (68). Compound 189 is one such inhibitor that targets the ATG12-ATG3 PPI interaction; it significantly reduces the number of autophagosomes and inhibits tumor growth in PC cells (69). Unfortunately, these inhibitors targeting autophagy-related proteins have not yet undergone clinical testing.

Interestingly enough, though, there are ongoing clinical trials combining traditional chemotherapy drugs or targeted therapy with autophagy inhibitors like HCQ plus FOLFOX (a CRC chemotherapy regimen) or bevacizumab bead sheet resistance, which may improve the anti-tumor effect on CRC patients (67, 70, 71). Additionally, HCQ plus atezolizumab combination therapy may also provide benefits for GIC precisely (70). In clinical practice for PC, the researchers did not observe any significant clinical significance of HCQ monotherapy. However, when combined with GEM therapy, HCQ treatment effectively improved median survival and disease-free survival in patients with PC (66). Radiotherapy is a crucial method in tumor treatment, and further exploration is needed to understand the therapeutic effect of autophagy inhibitors combined with radiotherapy in patients with cancer. Targeting biological molecules to regulate autophagy, such as the circRNA-autophagy axis described in this paper, represents a bold attempt to treat tumors.

The current field of autophagy is just a tiny fraction of the iceberg, with many issues still needing clarification. The same signaling pathway-mediated autophagy may play different roles in different cells in diseases such as cancer, inflammation, and neurodegeneration (72). CircRNA also mediates signaling pathways in this way (73). Studies have shown that elevated levels of circ_0032821 in GC cells inhibit autophagy, suggesting its significance as a critical regulator (74). Compared to other still emerging PCD, research on the impact of circRNA on autophagy is relatively mature, with numerous reports on the regulation of autophagy by circRNAs in GIC emerging in the past five years. There is currently no comprehensive discussion on the circRNA-autophagy axis in GIC, so it is necessary to study further the impact of autophagy on GIC under the regulation of circRNA.

## 2 Biological behavior of GIC

**Figure 3:**
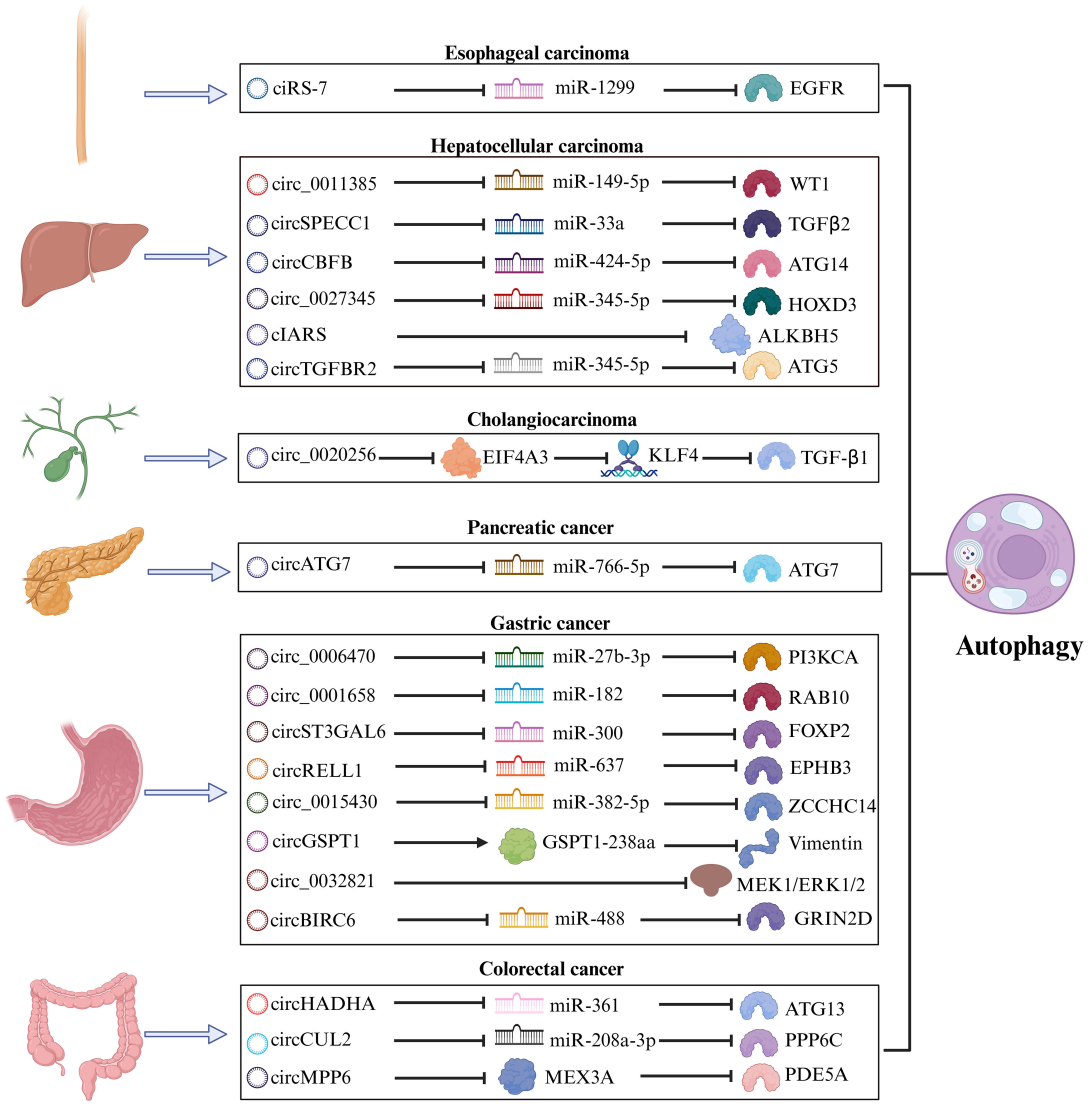
The mechanism of the circRNA-autophagy axis on biological activity. The mechanism by which circRNA mediates autophagy and impacts the progression of GIC is predominantly through the classical ceRNA pathway, thereby regulating the biological behavior of tumors. A minority of circRNA have been identified to interact with RBP or participate in translation processes, leading to autophagy-related alterations in tumor biology.

In this context, we emphasize the role of autophagy-related circRNA in GIC to better understand the functional roles of the circRNA-autophagy axis in different situations regarding the biological activity of GIC, such as proliferation, invasion, and metastasis (**Table 2**; **Figure 3**).

**Table 2:** The biological role of circRNA-autophagy axis and its potential biomarkers.

Cancer	CircRNA	Target	Biological activity	Role in autophagy	Clinical interest	ROC/ KM curve	Ref
GC	circ_0032821	MEK1/ERK1/2	promoting proliferation, migration, invasion	Resist	Diagnosis, Prognosis	KM curve, p=0.0001	(74)
	circ_0006470	miR-27b-3p/ PI3KCA	promoting proliferation, migration, invasion	Resist	Diagnosis	/	(75)
	circ_0001658	miR-182/RAB10	promoting proliferation; suppressing apoptosis	Induce	Diagnosis	/	(76)
	circST3GAL6	miR-300/FOXP2/MET	inhibiting proliferation, metastasis; inducing apoptosis	Induce	Diagnosis, Prognosis	KM curve, p=0.0315	(77)
	circBIRC6	miR-488/GRIN2D/ CAV1	promoting proliferation, migration, invasion	Resist	Prognosis, Treatment	/	(78)
	circGSPT1	GSPT1-238aa/vimentin/BECN1/14-3-3	inhibiting proliferation, migration, invasion	Resist	Diagnosis	/	(79)
	circRELL1	miR-637/EPHB3	inhibiting proliferation, migration, invasion; inducing apoptosis	Induce	Diagnosis, Prognosis	AUC=0.731, p=0.0025; KM curve, p=0.0092	(80)
	circRNA_15430	miR-382-5p/ZCCHC14	inhibiting proliferation, migration; inducing apoptosis	Induce	Diagnosis	/	(81)
CRC	circHADHA	miR-361/ATG13	inhibiting proliferation	Induce	Early diagnosis	/	(82)
	circMPP6	MEX3A/PDE5A	promoting proliferation	Resist	Prognosis	KM curve, p=0.0002	(83)
	circCUL2	miR-208a-3p/PPP6C	inhibiting proliferation; inducing apoptosis	Induce	Diagnosis	/	(84)
HCC	cIARS	ALKBH5	increasing SF-induced ferroptosis	Induce	Treatment	/	(85)
	circCBFB	miR-424-5p/ATG14	promoting proliferation	Induce	Diagnosis, Prognosis	KM curve, p=0.047	(86)
	circTGFBR2	miR-205-5p/ATG5	promoting proliferation; resisting apoptosis	Induce	Diagnosis, Prognosis	/	(87)
	circ_0027345	miR-345-5p/HOXD3	inhibiting proliferation, migration, invasion; inducing apoptosis under matrine treatment	Induce	Treatment	/	(88)
	circ_0011385	miR-149-5p/WT1	inhibiting proliferation, invasion; inducing apoptosis under aloin treatment	Induce	Treatment	/	(89)
CAA	circ_0020256	EIF4A3/KLF4/TGF-β1	promoting proliferation, migration, EMT	Resist	Diagnosis	/	(90)
PC	circATG7	miR-766-5p/ATG7	promoting proliferation, mobility	Induce	Diagnosis, Prognosis	AUC=0.728, p=0.0008; KM curve, p=0.0023	(91)
ESCC	ciRS-7	miR-876-5p/EGFR	/	Resist	Diagnosis	/	(92)

ROC, receiver operating characteristic; AUC, area under the curve.

### 2.1 GC

GC has consistently remained one of the most significant types of cancer worldwide, ranking as the fifth most prevalent malignant tumor with the fourth mortality rate globally (93). Due to the absence of specific early clinical indicators for GC, many patients are diagnosed at an advanced stage during endoscopy or imaging procedures. For individuals with advanced GC or distant metastasis, the 5-year survival rate is less than 5% (94). Urgent investigation into novel biomarkers and therapeutic targets for the early diagnosis and treatment of GC is essential. Numerous studies have revealed a significant correlation between circRNA and autophagy in the progression of GC (95).

Cui et al. proved that the circ_0006470/miR-27b-3p/PI3KCA axis regulates autophagy, proliferation, and invasion of GC cells. Specifically, circ_0006470 and PI3KCA act as negative regulators of autophagy levels while functioning as positive regulators of GC malignancy (75). Duan and colleagues discovered that circ_0001658 is highly expressed in GC cells and clinical specimens, promoting GC progression and autophagy by targeting miR-182/RAB10 (76). Silencing this circRNA inhibited the proliferation of GC cells and reduced IL3 site expression on GC cells through immunofluorescence analysis. Additionally, overexpression of circST3GAL6 was observed in both GC tissues and cells. This circRNA regulated downstream transcription factor FOXP2 through sponge miR-300 to inhibit the invasion of GC cells while promoting autophagy (77). Further investigation revealed that FOXP2 promotes autophagy by mediating the binding between circST3GAL6 and MET promoter to activate the AKT/mTOR pathway. The ultimate consequence of this mechanism is to augment the process of autophagy and thereby optimize the prognostic quality for patients diagnosed with GC. Tang et al. discovered that the circBIRC6-miR-488/GRIN2D axis synergistically promotes multiple malignant behaviors in GC. Specifically, circBIRC6 and GRIN2D exhibit concordant effects, as their upregulation reduces caveolin-1 (CAV1)-mediated autophagy (78).

Based on the latest findings, proteins derived from the translation of circRNA play a pivotal role in regulating various biological behaviors of tumors (96). The polypeptide GSPT1-238aa is encoded by circGSPT1. Hu et al. demonstrated that the circGSPT1 inhibits GC cells proliferation and lymph node metastasis, while its product GSPT1-238aa acts as an independent regulator to suppress malignant behavior in GC (79). Vimentin/BECN1/14-3-3 has been identified as an autophagy inhibitory complex, and GSPT1-238aa directly binds to Vimentin. Hu’s team extensively investigated the interaction between GSPT1-238aa and the autophagy inhibitory complex, revealing its efficacy in suppressing autophagy through the PI3K/AKT/mTOR pathway. The combined significance of circRNA translation products with autophagy in GC prognosis warrants further consideration.

Exosomes, secreted by cells into body fluids within organisms, encapsulate genetic materials from parental cells within multivesicular bodies and deliver them to recipient cells, including circRNA (97). Upon reaching recipient cells, exosomal circRNA can assume various functions. For instance, plasma exosomal circRELL1 derived from GC tissues has been confirmed to inhibit the proliferation and invasion of GC cells by mediating the miR-637/EPHB3 axis, in line with findings from cell and animal experiments involving circRELL1 (80). Inversely, the downregulation of exosomal circRELL1 led to a decrease in IL3 levels, suggesting that the circRELL1-autophagy axis also contributes to the biological behavior of GC cells. The implications of exosomal circRNA in cancer progression offer an additional avenue for early cancer diagnosis.

Liu et al. discovered that circ_0015430 exhibited downregulation in GC, facilitating GC cells proliferation and migration. Researchers found that circ_0015430 was also reduced in *Helicobacter pylori* (*H. pylori*)-infected chronic gastritis tissues and GC cells, accompanied by an increase in the abundance of autophagosome (81). Circ_0015430 regulated the dissemination process of GC cells and associated autophagy by acting as a sponge for miR-382-5p/ZCCHC14. The inhibition of circ_0015430 reversed the impact of *H. pylori* infection on autophagy. It is widely acknowledged that *H. pylori* infection induces chronic gastritis, serving as a common carcinogenic factor leading to a series of malignant pathologies in the gastric mucosa, according to the Correa cascade model (98). Therefore, circ_0015430 may be an effective therapeutic target for treating GC complicated with *H. pylori* infection.

### 2.2 CRC

The increasing prevalence of early-onset CRC has become a matter of growing concern. As per the data, approximately 11% of colon cancer patients and 23% of rectal cancer patients globally are anticipated to be under the age of 50 by 2030 (99). In chemotaxis in young individuals with CRC, unraveling more potent and dependable molecular biology markers, such as circRNA associated with the autophagy pathway, which is involved in the initiation and development of CRC (100).

Based on the evidence that serrated polyps can progress to CRC, researchers are increasingly persuaded that polyps play a significant role as precursor lesions in the development of CRC (101). The relevance of colonic polyps concerning colon cancer has become increasingly evident. Shi’s research team confirmed differential expression of circHADHA in the plasma samples from healthy individuals, patients with colonic polyps, and patients with colon cancer. Compared to healthy individuals, circHADHA was upregulated in the plasma of patients with colonic polyps and downregulated in the plasma of patients with colon cancer (82). Cell experiments demonstrated that circHADHA could inhibit the proliferation of colon cancer cells by enhancing autophagy. Dual-luciferase assay revealed that miR-361 could bind to circHADHA/ATG13, and overexpression of circHADHA could competitively bind to miR-361 to increase ATG13 and LC3-II levels for restoring autophagy. In summary, the dynamic changes observed in plasma circHADHA among patients with colon polyps and colon cancer provide valuable insights for the early detection of malignant tendencies associated with polyps. Additionally, targeting the circHADHA/miR-361/ATG13 axis may offer a novel biological approach to treating colon cancer.

As a biomolecule in RBP, MEX3A is significantly up-regulated in CRC, but its expression level is opposite to the autophagy marker LC3-II, indicating that MEX3A is an autophagy inhibitor. In subsequent experiments, researchers have further proved circMPP6, after forming complexes with MEX3A biological accumulation, can accelerate PDE5A mRNA degradation, MEX3A level and PDE5A levels of the rise of synergistic inhibition of autophagy and accelerate CRC malignant behavior (83). The competitive endogenous RNA (ceRNA) network represents a classical pathway through which circRNA regulates autophagy (102). In light of this, studies involving the circRNA-RBP complex are instrumental in comprehensively understanding the mechanism underlying the circRNA-autophagy axis.

To delve into the regulatory mechanism of circCUL2 in CRC, Yang et al. initially detected the downregulation of circCUL2 expression in collected CRC tissues. Subsequently, they ascertained that increased circCUL2 expression robustly inhibited CRC cell proliferation and arrested tumor growth in both Cell Counting Kit-8 (CCK-8) and tumor allograft experiments (84). Further experimental validation corroborated that circCUL2 induced autophagy through its interaction with miR-208a-3p, thereby exerting a tumor-suppressive role in CRC.

### 2.3 HCC

The emergence of HCC has garnered significant attention in the global healthcare community. At present, the primary etiology of HCC is transitioning from viral hepatitis to non-alcoholic fatty liver disease, a condition intimately linked with obesity (103). With the increasing burden of HCC, it is vital to identify novel biomarkers with enhanced sensitivity and specificity in addition to alpha fetoprotein (AFP), including circRNA, to guide the diagnosis and systemic treatment of HCC (104).

Sorafenib (SF) is the preferred systemic treatment for HCC and confers a survival benefit across different subtypes (105). CIARS, a highly expressed circRNA in HCC following SF treatment, interacts with ALKBH5 to antagonize the inhibitory effects on autophagy. Moreover, cIARS/ALKBH5 promotes ferroptosis in HCC cells after SF treatment (85).

Zhao et al. transfected circCBFB mimic into HCC cells and observed enhanced proliferation ability using CCK-8 assays, reversing the growth inhibition caused by an autophagy inhibitor called 3-methyladenine (3-MA) treatment (86). Furthermore, transfection of miR-424-5p mimic or ATG14 mimic led to decreased cell viability and inhibited autophagy in HCC cells. The investigator confirmed that circCBFB/miR-424/ATG14 enhances HCC proliferation activity by promoting autophagy via dual-luciferase assay. Therefore, there are promising prospects for the clinical application of circ-SPECC1 and circCBFB in improving the survival rate of patients with HCC. Wang et al. revealed that exosomal circTGFBR2, in combination with miR-205-5p, targets ATG5 downstream to enhance the autophagy process in HCC cells, thereby increasing their resistance to starvation stress (87).

Natural chemical products derived from Chinese herbal medicine exhibit anti-inflammatory, anti-tumor, and other therapeutic properties. Due to their relatively low toxicity and natural compounds, researchers have progressively investigated them. The mechanisms by which certain natural compounds, such as terpenoids, alkaloids, phenols, and flavonoids, inhibit tumor growth have been elucidated (106). Lin et al. experimented with Matrine to treat HCC and demonstrated its efficacy in inhibiting HCC progression. By observing epigenetic changes in the treatment group, they identified down-regulated circ_0027345 (88). Subsequent experiments revealed that Matrine utilized the circ_0027345/miR-345-5p/HOXD3 axis to promote autophagy while attenuating its anti-tumor effectiveness. On the other hand, Fu et al. identified an oncogene, circ_0011385, in aloin-treated HCC that targeted miR-149-5p/WT1. The administration of aloin effectively inhibited HCC cells proliferation and tumor growth. Inhibition of circ_0011385 expression was observed alongside WT1 inhibition, accompanied by increased autophagy markers LC3-II and BECN1 expression. Conversely, overexpression of circ_0011385 reversed the effects induced by aloin (89). Long-term regulation of circRNA can enhance the therapeutic effect of natural chemicals in treating HCC while also providing novel pharmacological mechanisms for future drug research and development.

### 2.4 Cholangiocarcinoma

The CAA can be classified into three types based on the location of the lesion: intrahepatic CAA, hilar CAA, and extrahepatic CAA. Although the incidence of CAA in GIC is relatively low, early detection through conventional tumor markers remains essential due to its high mortality rate (107). In recent years, dysregulated expression of circRNA has been implicated in the pathogenesis of CAA (108). EIF4A3 functions as an RBP that interacts with circ_0020256 to consolidate the expression of transcription factor KLF4 in CAA. This interaction further promotes TGF-β1 secretion (90), which plays a role in epithelial-mesenchymal transition (EMT) mediated by TGF-β1/Smad pathway activation (109). Furthermore, CAA cells activate cancer-associated fibroblasts (CAFs) via the TGF-β1/Smad pathway, while IL-6 secreted by CAF inhibits autophagy and accelerates CAA growth and infiltration. In future studies, targeting the circ_0020256/EIF4A3/KLF4/TGF-β1 axis could provide a novel approach for intervention in cholangiocarcinoma.

### 2.5 PC

PC is a markedly aggressive tumor, with pancreatic ductal adenocarcinoma being the most prevalent pathological type. The majority of patients are ineligible for surgical intervention upon diagnosis, and even after surgery, nearly three-quarters of patients experience recurrence within three years (110). He et al. identified a negative correlation between circATG7 and miR-766-5p but a positive correlation with ATG7 in PC. This circRNA was found to enhance invasion and metastasis in PC cells, upregulate ATG7 expression through miR-766-5p sequestration, and provide human antigen R protein (HuR) scaffold to stabilize ATG7 mRNA. Consequently, it can facilitate autophagy in PC cells (91). Recognizing contemporary medicine’s substantial challenges in diagnosing and treating PC is paramount. Consequently, circRNA is garnering increasing acknowledgment for its role in elucidating the pathogenesis of PC.

### 2.6 Esophageal carcinoma

Esophageal cancer is a prevalent tumor of the digestive tract, with squamous cell carcinoma (ESCC) and adenocarcinoma being the primary histological subtypes. As revealed by GLOBOCAN’s data, a projected increase of 58.4% in new cases and 61.7% in deaths due to esophageal cancer is anticipated from 2020 to 2040 (111). Meng et al. demonstrated that ciRS-7 exhibits abnormal expression in ESCC and functions as an oncogenic circRNA that regulates the miR-1299/EGFR axis, thereby impacting the AKT/mTOR pathway (92). CiRS-7 acts as a negative regulator of autophagy, suggesting that the integrated ciRS-7-autophagy axis may contribute to enhancing the malignant behavior of ESCC.

## 3 Drug resistance

**Figure 4:**
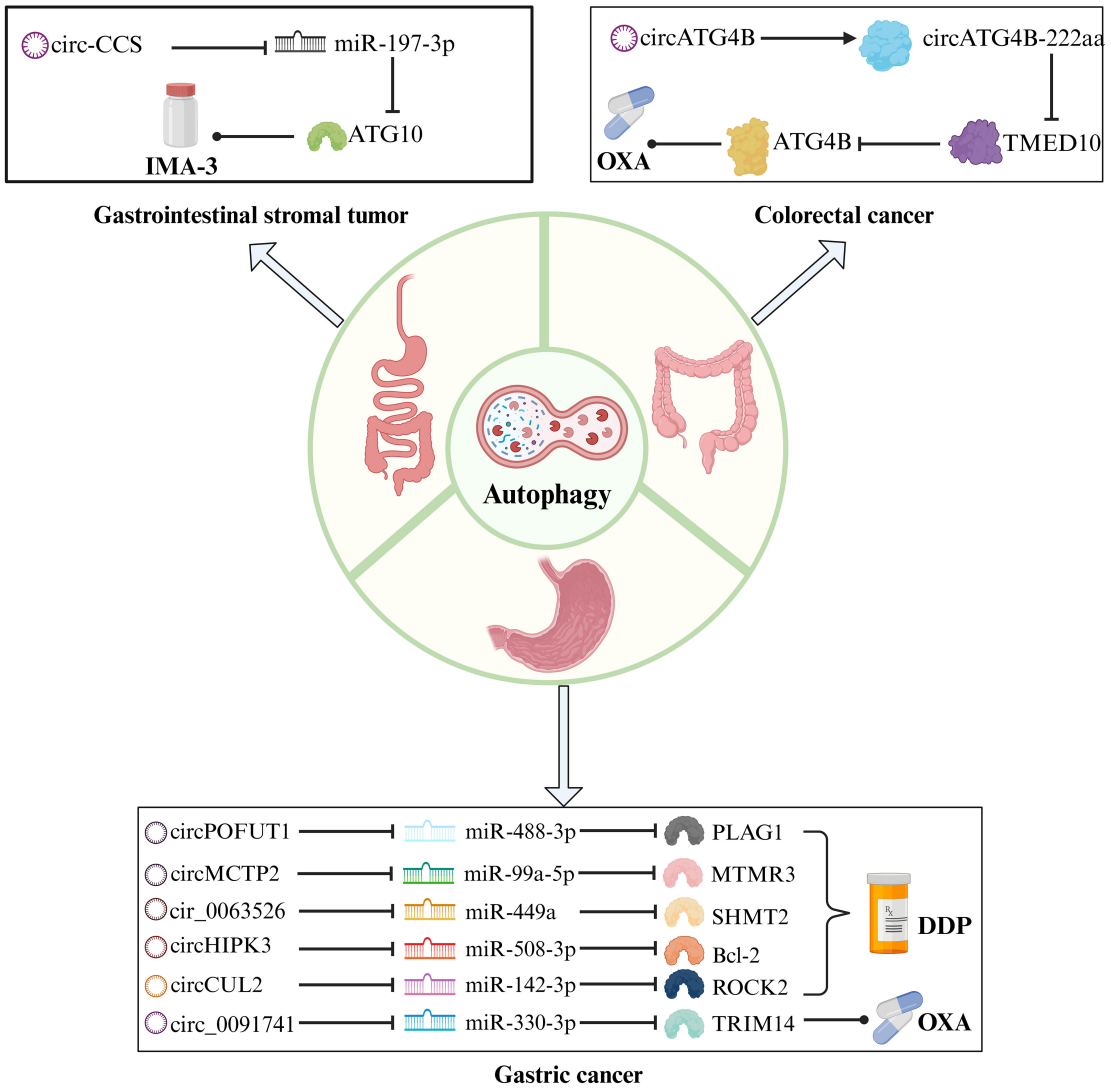
The mechanism of the circRNA-autophagy axis on drug efficacy. CircRNA-mediated autophagy regulates the signaling pathway of chemoresistance. Platinum drugs such as DDP and OXA are traditional chemotherapy drugs for the treatment of GC and CRC, and IMA-3 is a common drug for the treatment of GIST.

Pharmaceuticals assume a vital function in cancer management, acting as indispensable instruments to mitigate preoperative lesions, forestall postoperative recurrence, and offer palliative care for advanced stages. The conventional agents employed in GIC therapy primarily encompass DPP, carboplatin, oxaliplatin (OXA), and GEM. The advent of targeted drugs and anti-programmed death ligand 1 (PD-L1) drugs has opened up new opportunities for patients with GIC. However, drug resistance is a common challenge encountered in GIC treatment regardless of the specific antineoplastic agent employed. Scientists have elucidated various mechanisms underlying tumor drug resistance, including reduced intracellular drug concentration, disrupted drug metabolism processes, target mutations or difficulties in forming effective drug-target complexes, and alterations in signaling pathways (112). We actively explore new biomarkers that may regulate drug resistance and have identified the research potential of the circRNA-autophagy axis (**Table 3**; **Figure 4**), as well as circRNA and autophagy-related factors as promising biological targets to enhance the therapeutic efficacy of GIC medications.

**Table 3:** CircRNA-autophagy axis mediated drug resistance and its potential biomarkers.

Cancer	CircRNA	Target	Drug efficacy	Autophagy	Clinical Interest	ROC/ KM curve	Ref
GC	circPOFUT1	miR-488-3p/PLAG1/ ATG12	DDP resistance	Induce	Diagnosis, Prognosis, Treatment	KM curve, p=0.046	(113)
	circMCTP2	miR-99a-5p/ MTMR3	DDP sensitivity	Resist	Diagnosis, Prognosis, Treatment	AUC=0.945 KM curve, p=0.0088	(114)
	circ_0091741	miR-330-3p/TRIM14/ Dvl2/Wnt/β-catenin	OXA resistance	Induce	Diagnosis, Treatment	/	(115)
	circ_0063526	miR-449a/ SHMT2	DDP resistance	Induce	Diagnosis, Treatment	/	(116)
	circHIPK3	miR-508-3p/Bcl-2/BCEN1/SLC7A11	DDP resistance	Resist	Diagnosis, Treatment	/	(117)
	circCUL2	miR-142-3p/ROCK2	DDP sensitivity	Resist	Diagnosis, Prognosis, Treatment	AUC=0.79, P<0.001 KM curve, p=0.0132	(118)
CRC	circATG4B	circATG4B-222aa/ TMED10/ATG4B	OXA resistance	Induce	Diagnosis, Prognosis, Treatment	/	(119)
GIST	circ-CCS	miR-197-3p/ATG10	IMA-3 resistance	Induce	Diagnosis, Treatment	/	(120)

ROC, receiver operating characteristic; AUC, area under the curve.

### 3.1 GC

Luo et al. examined the function of circPOFUT1 as a ceRNA sponge for miR-488-3p, and identified PLAG1 as a protooncogene that directly interacts with miR-488-3p to regulate ATG12 expression in GC indirectly (113). The upregulation of circPOFUT1 in GC promotes tumor progression, autophagy progression, and confers resistance to DPP through autophagy-related mechanisms. Numerous studies have centered on DPP resistance. Sun et al. also demonstrated that circMCTP2, which modulates DPP resistance, collaborates with miR-99a-5p/MTMR3 to suppress the autophagic behavior of DPP-resistant GC cells (114). The functional significance of circMCTP2 in GC is dissimilar to that of circPOFUT1, as it serves to restrain DPP resistance.

Exosomal circRNA regulates the malignant behavior of tumors and serves as a novel mechanism of cancer drug resistance (121). circ_0091741 (115) and cir_0063526 (116) facilitate intercellular communication through exosomes, promoting autophagy-induced OXA and DPP resistance via the miR-330-3p/TRIM14 axis and the miR-449a/SHMT2 axis, respectively. These exosomal circRNAs effectively propagate drug resistance among cells. Notably, increased expression of TRIM14 activates the Dvl2/Wnt/β-catenin signaling pathway to enhance OXA resistance in GC, while elevated expression of SHMT2 promotes DPP resistance. Surprisingly, Shang et al. confirmed that the miR-508-3p/Bcl-2/BECN1/SLC7A11 axis negatively regulates autophagy and ferroptosis, where autophagy activates ferroptosis (117). Considering that serum levels of exosomal circHIPK3 were lower in DPP-sensitive patients compared to DPP-resistant patients, this circRNA’s role in enhancing DPP resistance was validated. The findings suggest a notable reduction in serum exosomal circHIPK3 levels in GC patients treated with DPP. The exosomal circ_0091741, circ_0063526, and circHIPK3 molecules can serve as robust, non-invasive indicators for monitoring the efficacy of chemotherapy in GC.

### 3.2 CRC

CircRNA plays a diverse function in CRC, including protein translation (122). ATG4B is a vital signaling molecule that governs the autophagy transduction pathway, and its activity is intricately linked to the high mortality rate and chemoresistance of CRC (123). CircATG4B is a circRNA generated through processing and splicing of the ATG4B gene. Pan et al. contrasted OXA-resistant CRC cells with OXA-sensitive CRC cells, corroborating a significant expression of circATG4B and its protein coding product, cirCATG4B-222aa, in resistant cells (119). Regarding autophagy, circATG4B-222aa functions as a binding platform for TMED10 to augment autophagy. TMED10 is recognized to regulate ATG4B as an autophagy regulator negatively (124). Upon binding to TMED10, circATG4B-222aa dissociates from it and releases more ATG4B, thereby attenuating the anti-tumor effect of OXA in CRC. A principal challenge resides in translating circATG4B-222aa into clinical practice.

### 3.3 Gastrointestinal stromal tumor

The primary origin of GIST is spontaneous mutations in receptor tyrosine kinase (KIT) or platelet-derived growth factor α (PDGFRα). Tyrosine kinase inhibitors and surgical resection are prevalent treatment methods for GIST (125). Sui et al. detected elevated expression of circ-CCS in patients with imatinib (IMA-3) resistance and recurrence, which promotes the proliferation, dissemination, and autophagy of GIST cells (120).miR-197-3p binds to circ-CCS to regulate ATG10, ultimately resulting in IMA-3 resistance. IMA-3 resistance poses a substantial challenge in the management of GIST. By investigating circ-CCS, potential breakthroughs in drug resistance may be uncovered.

## 4 The future directions of autophagy-related circRNA

The emergence of immunotherapy (IT), a significant milestone in cancer treatment, encompasses four main types: immune checkpoint inhibitors (ICIs), with PD-L1 being the classic ICIs, peripatetic immunotherapy, tumor vaccines, and chimeric antigen receptor T-cell therapy (126). Autophagy-related molecules stimulate immune responses to engage in anti-tumor therapy and recruit immune cells such as natural killer cells and T lymphocytes to target tumor cells (127). Studies have demonstrated that ATG5 and ATG7 regulate ferroptosis-mediated tumor IT (127). Taraborrelli et al. reported that ATG16L1 protects CRC from immune cell infiltration and cytokine-induced inflammation (128).

In addition, the proteins encoded by circRNA play significant roles in the field of biological vaccines (129). Lipid nanoparticles serve as a delivery medium to transport circRNA into cells, while the protein acts as a specific antigen to initiate systemic immune response (130). A novel engineered circular mRNA vaccine introduces immune cells into tumors and inhibits tumor growth. Utilizing engineered circRNA enhances translation efficiency and protein stability while reducing immunogenicity (131). Although there have been numerous reports on autophagy or circRNA research and their applications in IT, studying the circRNA-autophagy axis in IT is still early. Exploring the connection between the circRNA-autophagy axis and IT will greatly improve cancer treatment.

Immune evasion is a challenge in cancer treatment. When the expression of mitochondrial translocator protein (TPSO) was specifically increased in HCC, autophagy-mediated ferroptosis was inhibited. Mechanistically, upregulated TPSO targeted the p62/KEAP1/Nrf2 pathway and upregulated the expression of PD-L1, thereby promoting the immune evasion of HCC cells (132). Miao et al. reported that circ_0136666 combined with miR-375/PRKDC axis and phosphorylation PD-L1 improved immune evasion GC cells (133). PD-L1 and MHC-I/II regulation are important in immune evasion (134). A comprehensive understanding of the mechanism of autophagy and circRNA in immune evasion can better avoid the treatment risks caused by immune evasion.

## 5 Discussion

The regulation of autophagy by circRNA has been associated with the modulation of tumor progression, cardiovascular diseases, and neurodegenerative disorders (55). GIC represents a significant form of cancer. In the long run, the circRNA-autophagy axis presents a novel perspective for diagnostic and therapeutic strategies targeting GIC.

Indeed, the same circRNA plays a principal role in various human tumors (135). By carefully comparing the aberrantly expressed circRNA in different tumor types, it has been observed that they not only regulate the progression and drug resistance of multiple tumors, but also participate in the process of autophagy. CircCUL2 and circHIPK3 act as miRNA sponges to target downstream genes, forming a circRNA-autophagy axis that inhibits CRC progression (84) and promotes GC DPP resistance (117), respectively. Previously, Peng et al. demonstrated that circCUL2 mediates the inhibition of autophagy through the miR-142-3p/ROCK2 axis to induce DPP sensitivity in GC (118). Expanding beyond GIC, Wang et al. confirmed that circHIPK3 binds to and inhibits VCP (a kind of RBP), thereby suppressing BECN1-mediated autophagy and impeding bladder cancer progression (136). Consequently, the regulatory roles of both the circCUL2-autophagy axis and circHIPK3-autophagy axis have been elucidated across different tumor types, thus highlighting the need for further exploration of the involvement of the circRNA-autophagy axis in additional malignancies.

In reality, the development of new anti-tumor drugs is imminent. Natural chemicals derived from plants impact HCC progression by modulating autophagy through circRNA (89), which holds great promise. The liver serves as the primary site for anthocyanin metabolism, and Matboli’s team has validated certain anthocyanins like cyanidin-3-glucoside (C3G) to exhibit a dose-dependent anti-liver cancer effect via cell cycle regulation (137). Zabady et al. administered C3G to rats induced with precancerous lesions. Compared to the C3G treatment group, the precancerous lesion group showed increased levels of AFP and liver enzymes and decreased albumin content, consistent with Matboli’s experimental findings (138). Even more striking, circ_0001345 and autophagy-related gene ATG16L1 were concurrently upregulated in the C3G treatment group, while miRNA-106b was downregulated upon C3G treatment. Therefore, it was predicted that C3G could enhance ATG16L1-mediated autophagy through the circ_0001345/miR-106b/ATG16L1 axis identified via database analysis to achieve therapeutic goals for HCC treatment. Yang et al. isolated total flavonoids, luteolin, and apigenin from Scutellaria barbata D.Donc (SB) and Oldenlandia diffusa (Willd.) Roxb (OD) demonstrates their ability to inhibit the invasion of HCC cells, suppress hepatitis B virus replication activity, and hinder autophagy processes. The HCC cells treated with SB extract (SBE) and OD extract (ODE) exhibited a diverse range of differentially expressed circRNA. Researchers constructed a circRNA-miRNA-gene network diagram associated with the SBE and ODE. At the same time, further investigation is required regarding the involvement of the circRNA-autophagy axis in SBE and ODE (139). In future studies, combining traditional chemical drugs with natural compounds will greatly contribute to HCC treatment advancements and expand their application beyond HCC into GIC treatment.

The metabolic microenvironment significantly influences the metastatic dissemination of cancer. Altered metabolic status in the microenvironment leads to changes in nutrient uptake and oxidative stress. Cancer cells employ the autophagy pathway as an adaptive response to their surrounding environment, thereby regulating their ability to metastasize to distant organs (140). Gastrointestinal organs are commonly affected sites of metastasis; for instance, the liver is pivotal in tumor metastasis (141). Wang et al. confirmed that circROBO1 and FUS were highly expressed in BC, with KLF5-FUS promoting circROBO1 back-splicing. This behavior further facilitated BC cells proliferation, metastasis, and tumor growth (142). Subsequent experiments verified that miR-217-5p counteracted the malignant events induced by circROBO1 and formed a signaling pathway with KLF5 to establish the circROBO1/miR-217-5p/KLF5 axis. This axis inhibited afadin-related autophagy while promoting liver metastasis of BC through positive feedback mechanisms. Therefore, the circRNA-autophagy axis also regulates secondary GIC, and understanding its regulatory mechanism may improve prognoses for primary cancers.

The exact contribution of autophagy to the biological behavior and drug resistance of GIC remains to be fully understood. While it has been demonstrated that circFARP1 in PC plays a role in CAF and induces GEM resistance, the interaction between autophagy and circFARP1 remains unclear (143). Previous studies have shown that the TGF-β1/Smad2/3 pathway upregulates ATG4 levels, leading to reduced efficacy of GEM in PC (144). Furthermore, activation of the TGF-β1/Smad pathway induces CAF activation in CAA and promotes the secretion of autophagy-related mediators (90). Therefore, the potential link between circFARP1 and the autophagy pathway in PC warrants further investigation. ULK1 is a known activator of autophagy (145). Wang et al. demonstrated that ULK1, as a downstream target of miR-142-5p, was positively regulated by circTMEM87A to enhance proliferation, metastasis, and invasion of GC cells. However, this study did not assess changes in BECN1, IL3-II, or p65 levels (146). It is hypothesized that circTMEM87A may target ULK1 and participate in regulating autophagy processes. Importantly, it should be noted that ULK1 also possesses non-autophagic functions.

Autophagy-related circRNA accurately target autophagy genes or pathways during the process of autophagy and regulate cancer progression. CircRNA are widely detected in human body fluids (7). Furthermore, its inherent stability aids in validating the clinical significance of more autophagy-related circRNA in cancer (121), establishing a comprehensive database for subsequent clinical applications. The expression of circRNA varies among different patients and disease stages (147). Therefore, personalized circRNA treatments based on individual differences will be more targeted. Biological nanoparticles and viruses serve as carriers to encapsulate autophagy-related circRNA and deliver them to target cells, protecting against degradation caused by external stressors and promoting production (148, 149). The pre-clinical trials should be rigorously regulated under ethical and moral guidelines, with a focus on minimizing drug toxicity and side effects, as well as optimizing research and development costs for the benefit of a larger population of cancer patients.

## 6 Conclusions

This review summarizes recent studies on the circRNA-autophagy axis in the biological behavior and drug resistance of GIC. CircRNA regulates autophagy through ceRNA, RBP binding, and other mechanisms within multiple signaling pathways. Emerging research highlights the interplay between circRNA translation function and autophagy, influencing the progression and resistance of GIC, including secondary GIC. The complexity of autophagy in GIC necessitates further investigation due to its dual nature. Several circRNA have been identified as regulators of autophagy in various types of GIC and other cancers. The generalizability of the circRNA-autophagy axis beyond individual GIC to encompass a broader range of cancers or cancer screening applications merits exploration. Notably, plant extracts have demonstrated involvement with the circRNA-autophagy axis in treating GIC, highlighting its potential for application in novel drug development. Future research will primarily focus on elucidating the specific mechanism of the circRNA-autophagy axis in regulating the occurrence and progression of GIC. This endeavor aims to utilize the circRNA-autophagy axis as a novel biological marker for early diagnosis and severity evaluation of GIC. It also explores its potential as a therapeutic target to overcome drug resistance and reduce the global disease burden associated with GIC.
